# A Hardware-Based Configurable Algorithm for Eye Blink Signal Detection Using a Single-Channel BCI Headset

**DOI:** 10.3390/s23115339

**Published:** 2023-06-05

**Authors:** Rafael López-Ahumada, Raúl Jiménez-Naharro, Fernando Gómez-Bravo

**Affiliations:** 1 Departamento de Ingeniería Electrónica Sistemas Informáticos y Automática, Universidad de Huelva, 21007 Huelva, Spain; 2 Centro Científico Tecnológico de Huelva (CCTH), University of Huelva, 21007 Huelva, Spain

**Keywords:** eye blinking detection, FPGA, Arduino, Mindwave headset

## Abstract

Eye blink artifacts in electroencephalographic (EEG) signals have been used in multiple applications as an effective method for human–computer interaction. Hence, an effective and low-cost blinking detection method would be an invaluable aid for the development of this technology. A configurable hardware algorithm, described using hardware description language, for eye blink detection based on EEG signals from a one-channel brain–computer interface (BCI) headset was developed and implemented, showing better performance in terms of effectiveness and detection time than manufacturer-provided software.

## 1. Introduction

The detection of eye blinking remains relevant in the literature, where two distinct motivations can be found. The first motivation involves the elimination and/or adaptation of electroencephalographic (EEG) signals, since the presence of artifacts renders such signals unusable for drawing conclusions (e.g., diagnoses). In the former case, the data set that needs to be processed is reduced, and in the latter case, the quality of the data set to be processed is improved [1,2,3]. These studies typically include healthy individuals as well as those with some kind of pathology. The second motivation involves assigning a specific task to the presence of blinking. Generally, the assigned task is a confirmation task. Applications can vary widely, ranging from applications targeted at people with disabilities, such as for the control of electric wheelchairs, to security applications, such as identifying a dangerous situation [4,5].

Eye blinking detection algorithms are generally based on image recognition algorithms and, therefore, intensive mathematical calculations are required [6,7,8]. As such, these algorithms are implemented in microprocessor- or microcontroller-based systems. However, the use of these systems entails higher energy consumption and lower latency. One way to optimize these parameters is to use other systems with improved characteristics. An example of such a system that can improve microprocessor performance is a field-programmable gate array (FPGA) device.

Using FPGA devices can significantly enhance system performance by enabling the implementation of concurrent systems. FPGA devices are composed of configuration logic blocks, input–output blocks, and a connection array. Depending on the model of the FPGA device, various cores, such as random access memory (RAM) blocks, media access control (MAC) units, and microcontrollers, may also be included. This architecture allows for the implementation of system-on-chip (SoC) designs that combine both software and hardware elements within a single FPGA device, making the implementation of the desired behavior much easier.

The popularity of FPGA devices has been increasing across many application fields thanks to their ability to implement SoCs with software and hardware components in a single device. Furthermore, FPGA devices allow for greater concurrency, which can enhance the performance of eye blink detection algorithms.

In most studies, the goal is to efficiently detect and eliminate signal artifacts. In our case, we aimed to process the signal quickly and effectively to use eye blinking as control signals for human–machine interfaces (HMIs), detecting single, double, triple, and even quadruple intentional blinks. Along with this, we also sought to adequately measure the strength or intensity of these blinks since proper training allows a person to use this difference as a control signal too.

In this article, we propose the implementation of a real-time algorithm for blink detection based on artifact detection in EEG signals using an FPGA device. The signals come from a single-channel brain–computer interface, and the integration of the entire system allows for efficient power consumption and latency results compared to a commercial platform.

The article is structured as follows. After this introduction, in Section 2, we review some of the recent literature about eye blink detection and the use of FPGA devices. In Section 3, we describe the proposed architecture by analyzing the signal to be detected. In Section 4, we describe the testing of the proposed environment, choosing the best strategy that allowed for correct artifact detection. In Section 5, we analyze the results with a battery of different scenarios. Finally, in Section 6, we draw some conclusions.

## 2. Related Work

The detection of eye blinks is currently applied as a human–machine interface (HMI) in many areas of application [9,10]. For instance, in the automotive field, it is used as a safety measure for detecting fatigue [11,12,13] and to prevent possible accidents [14,15]. This strategy is applied either to activate a driver warning system or to trigger an automatic braking mechanism. In the home automation field, eye blinking detection is used to confirm a certain decision [16,17,18]. In medical applications, it is applied to make life easier for paralyzed patients by substituting more traditional interactions (such as the use of hands) with the eye blinks in order to activate certain systems (for example, controlling a wheelchair [19,20]). In addition, it is used to aid in diagnosis (for example, to distinguish between Alzheimer’s patients and non-affected older individuals [21]).

The detection mechanisms can be grouped into two categories [22]. The first mechanism is based on image processing using data obtained by a camera [4,23,24]. This solution includes a structure to place a camera pointing at the user’s face [25,26]. However, this structure is not always suitable, depending on the system to be controlled. This is more significant when the user suffers from a disease that keeps them from moving. All of these proposals use some type of machine learning for image processing, but this can limit the range of applications to which they can be applied.

The second mechanism is based on the interference produced by blinking in EEG signals. In the domain of EEG signals, an artifact is any behavior that does not originate from the brain, such as eye blinks, muscle movements in the face, or disturbances caused by the heart (pulse artifacts). Processing EEG measurement data in order to eliminate such interferences can be complicated but has been satisfactory to date [27,28].

The artifact is observed in the EEG signal. Evidently, in the case of BCIs with multiple electrodes, the electrodes with signals showing the most interference (and, therefore, allowing more straightforward detection) are the frontal electrodes, corresponding to the FP1 and FP2 positions of the international 10–20 system. Therefore, studies focus on the waves generated in the electrodes in these positions. Although the signals returned by the EEG are raw signals, the processing is usually performed with some type of feature derived from these signals [1,2]. The most significant examples of these features are as follows:

- Kurtosis, which can be seen as the shape of the peak of the wave. Blink detection implies that this peak is narrow and pronounced, so it has a high value;
- Skewness, indicating the symmetry or asymmetry of the wave. Blink detection implies that the wave is very symmetrical, so it has a value very close to zero;
- Signal variance. Blink detection implies a high value;
- Standard deviation. Detection implies a high value;
- Peak-to-peak amplitude. Detection implies a high value;
- Maximum value. Detection implies a high value;
- Minimum value. Detection implies a high value;
- Mean value. Detection implies a high value;
- Normality test (determines how a distribution fits a normal distribution, indicating randomness). Detection indicates a low value;
- Entropy. Blinking decreases entropy because it is a signal;
- Scalp topography, measuring differences in signals from different electrodes.

Detection involves choosing a threshold for the above features that determines the presence of a blink. These methods are the simplest and fastest [3]. The main problem with these methods is the correct choice of the threshold used.

A second methodology consists of using learning algorithms to detect the appropriate forms in the above figures [3,5,29]. The main problem with these methods is the high computational load.

Finally, it is worth noting the platform on which these detection mechanisms are implemented. In most cases, this platform is a computer using suitable software, mainly Matlab (MathWoks, Natick, MA, USA), although specific programs implemented in C or Python (Python Software Foundation, Wilmington, DE, USA) have also been used. However, when the application requires real-time operation and/or has strict consumption requirements, the chosen platform is an FPGA [30,31,32], either as a co-processor to aid a computer or as a single implementation platform. FPGA-based platforms provide improvements in power consumption, as well as system performance, by taking advantage of module concurrency and their specific character, as already mentioned in the introduction.

## 3. System Architecture and Characterization of Eye Blink Signals

**Figure 1:**
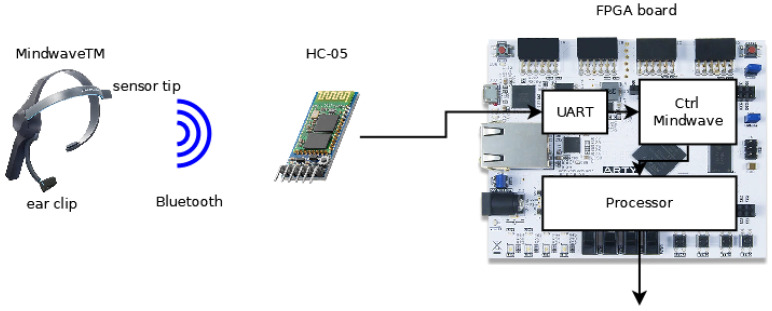
Architecture of the proposed system.

The architecture of the system implemented is shown in Figure 1 and consists of the following elements: a BCI (Mindwave™ headset, San Jose, CA, USA, [33]) capturing the EEG signals, an HC-05 Bluetooth module [34] allowing the interface to communicate with the FPGA device, and an FPGA device included in the Artix-7 35T evaluation kit (Xilinx, San Jose, CA, USA) [35]. The modules implemented in the FPGA device were a universal asynchronous receiver–transmitter (UART) module, receiving data from the Bluetooth module; the mindset communication controller, decoding the protocol of the headset; and the processor, processing the data received.

**Figure 2:**
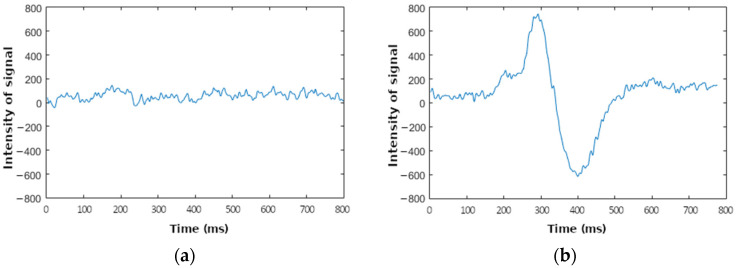
Example epochs containing two different waveforms obtained using Mindwave headset. (**a**) Waveform without eye blinking. (**b**) Waveform with eye blinking.

The first step is to characterize the waveform associated with a blink that must be recognized. Data from the waveform were obtained as follows. The EEG signal was obtained from the headset by decoding the Neurosky protocol to obtain raw values. Finally, these values were plotted by using Matlab^TM^ in two different situations: with and without eye blinking (see Figure 2).

A comparison of both waveforms demonstrates the feasibility of the detection of eye blinks from EEG signals because a low-frequency and high-amplitude wave appears with the blink. This wave, produced by an action different from brain activity, is known as an artifact. Considering that brain activity is due to the performance of a specific task that requires a certain level of concentration, which is the objective we pursue, the characteristics of this wave are as follows:

- It is a wave over a slightly variable DC level different from zero;
- Its amplitude is higher than the amplitude of waves due to brain activity;
- Its frequency is lower than the frequency of waves due to brain activity.

**Figure 3:**
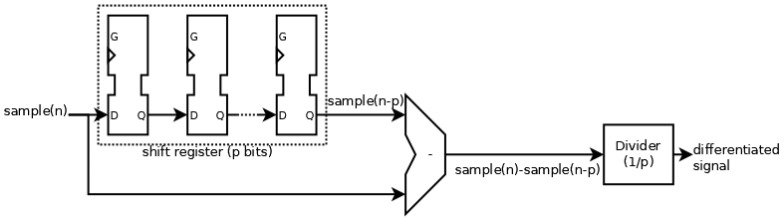
Scheme of the differentiator implementation.

The system to detect this artifact must have a differentiator to eliminate the DC value, and a low-pass filter to eliminate the higher-frequency wave due to brain activity. Equation (1) shows the implementation of the differentiator as an approximation of its definition.

(1) $$F^{\prime }\left(x\right)=\underset{x\to x_{0}}{\lim}\frac{f\left(x\right)-f\left(x_{0}\right)}{x-x_{0}}≅\frac{f\left(x\right)-f\left(x_{0}\right)}{x-x_{0}}\;F^{\prime }\left[n\right]=\frac{F\left[n\right]-F\left[n-p\right]}{p}$$

where *F*[*n*] is the current sample and *F*[*n* − *p*] is the *p*-th preceding sample. The differentiator also has behavior similar to that of a low-pass filter. The cut frequency is lower at a higher *p*. Therefore, both behaviors can be obtained with a differentiator. The implementation used is shown in Figure 3. This implementation consists of an FIFO memory (used as a shift register) to obtain the two samples, subtraction to obtain the difference, and a divider. In order to reduce the necessary hardware resources, p is a potential of 2 and, hence, the implementation of the divider is simply produced by a shift.

**Figure 4:**
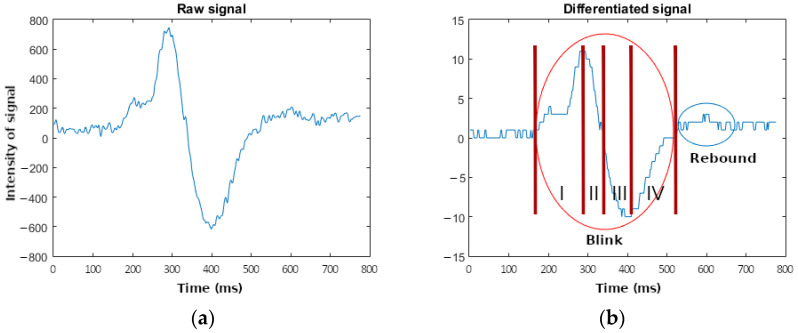
Comparison between the wave of the raw signal (**a**) and the wave of the differentiated signal (**b**) corresponding to an artifact (indicating the different zones used in the proposed algorithm) and a rebound. Zone I the algorithm seeks the maximum value, zone II, the algorithm seeks the edge between the maximum and minimum values, zone III, the algorithm seeks the minimum value and zone IV, it seeks the end of the artifact.

The number p is chosen to be 64 because a low-pass filter with the adequate cut frequency (below 4 Hz) requires 61 samples [36]. Figure 4 shows the waveform due to the blink of the raw signal (Figure 4a) and the differentiated signal (Figure 4b). In Figure 4b, it can be seen that, despite eliminating the DC value (the oscillation is between 10 and −10), the waveform remains. The main wave is marked in red, and it has the same frequency as the original wave. However, an extra oscillation appears, marked in blue in Figure 4b, due to the rebound of the main wave. The detection algorithm should detect the main wave and discard this additional oscillation, which could be misinterpreted as a new blink.

**Figure 5:**
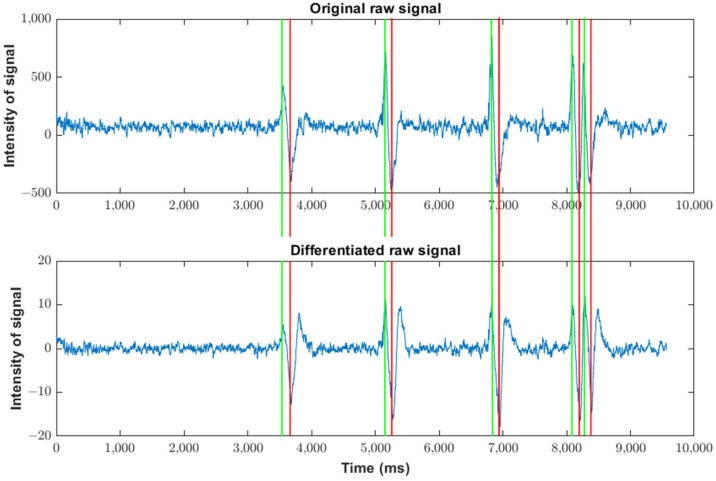
Comparison between samples of raw and differentiated signals corresponding to several artifacts. Green (or red) lines correspond to the samples with the maximum (or minimum) values in the differentiated signal.

It is remarkable that the samples corresponding to the peaks of the differentiated signal are similar to those of the raw signal. The samples corresponding to five artifacts (marked p1 to p5) are shown in Figure 5. Green (or red) lines correspond to the samples with the maximum (or minimum) values in the differentiated signal. It can be seen that the maximum and minimum values of the differentiated and the raw signals appear very close in time. In fact, the sample indexes corresponding to the maximum and minimum values of both raw and differentiated signals are very similar. The relative difference between them is lower than 0.7% and, hence, the index of the differentiated signal can be used to estimate the position of the maximum or minimum value in the raw signal and to obtain a value related to the strength of the blink.

## 4. System Implementation

The following step involves the implementation of the system included in the FPGA device. This system is made up of an UART module, a controller of the mindset communication protocol, and an algorithm for eye blinking detection.

### 4.1. Mindset Communication Protocol Controller

**Figure 6:**
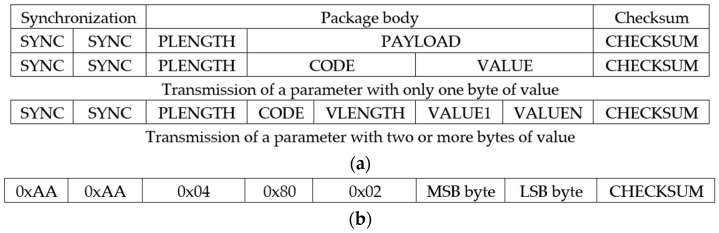
Structure of thinkgear packages. (**a**) Structure of a generic package. (**b**) Structure of a package corresponding to a raw signal.

The mindset communication protocol [37] involves the use of thinkgear packages. Its structure, shown in Figure 6a, involves three parts: the synchronization to identify the start of the package consisting of two bytes 0xAA, the package body to identify the significance of the package composed of the length of the package (PLENGTH) in the number of bytes and the payload, and, finally, the checksum to identify possible errors in the transmission. The structure of the payload differs depending on the code transmitted. When the code is lower than 0x80 (the number of bytes is 1), the payload structure consists of the code and its value, whereas if the code is not less than 0x80 (the number of bytes is more than 1), then the payload structure consists of the code, the value length (VLENGTH), and the values of the required bytes. In Figure 6b, the structure of a raw signal package is shown. It is a package with a value that has two bytes starting from the most significant byte.

**Figure 7:**
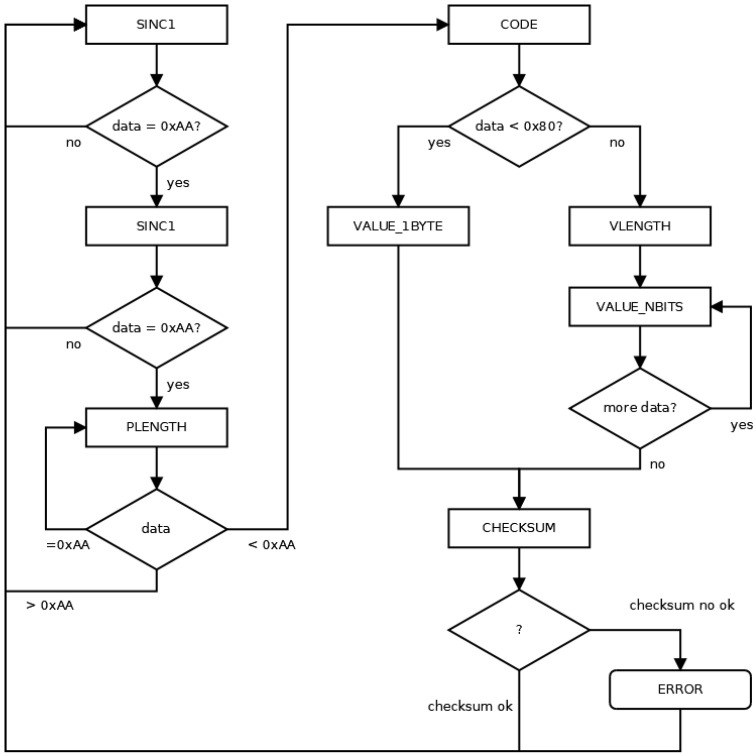
Behavior of the controller of the mindset communication protocol.

The behavior during the implementation of the controller to manage this protocol is shown in Figure 7, where changes occur with the arrival of data through the UART module. The behavior is based on managing the arrivals of the packets shown in Figure 6. When the complete packet is received, the value of the CHECKSUM data is checked. If it is correct, the received value is stored in a buffer (reg), while if the value is incorrect, an error message is sent. The reg buffer is addressed with the possible values of the signal code, so the value is stored in the reg address corresponding to its code. In the particular case of the raw signal, the addresses are 0x80 (MSB byte) and 0x81 (LSB byte).

### 4.2. Eye Blinking Detection Algorithm

**Figure 8:**
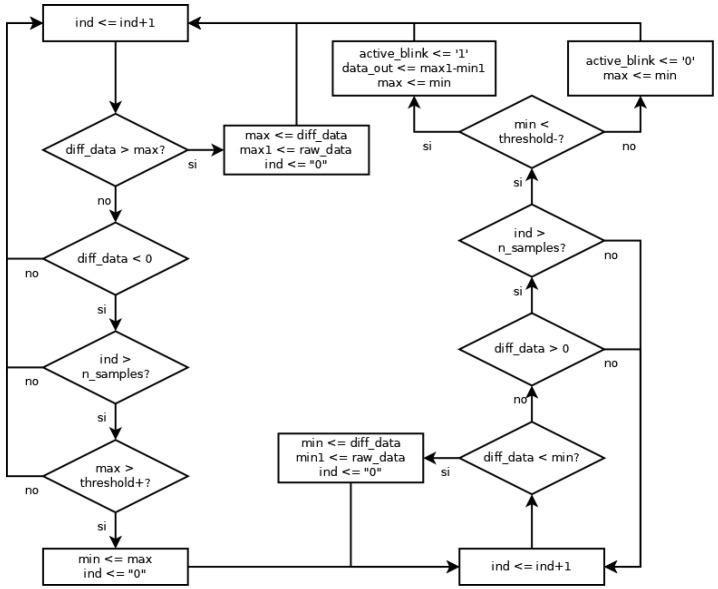
Behavior of the algorithm for eye blinking detection.

Finally, the algorithm for eye blinking detection uses the differentiated values shown in Figure 4. This algorithm seeks the maximum and minimum values in the same slope according to that figure. The behavior of the detection is shown in Figure 8. Firstly, the algorithm seeks a maximum value in the differentiated signal (diff_data). This maximum value involves certain characteristics:

- It must be higher than 0;
- It must be higher than a certain threshold (threshold+). This threshold determines the sensitivity of the algorithm;
- It must be maintained across a significant number of samples (n_samples), avoiding the high-frequency changes.

When a maximum value is detected, the algorithm seeks a minimum value in the differentiated signal. This new search is similar, and the minimum value should check for equivalent features:

- It must be lower than 0;
- It must be lower than a certain threshold (threshold-). This threshold determines the sensitivity of the algorithm;
- It must be maintained across a significant number of samples (n_samples), avoiding the high-frequency changes.

Once the maximum and minimum values have been detected, the algorithm identifies an eye blink. The value assigned to the blink is the amplitude of the raw signal because the samples of the maximum and minimum differentiated signals are very similar to the raw signal.

**Figure 9:**
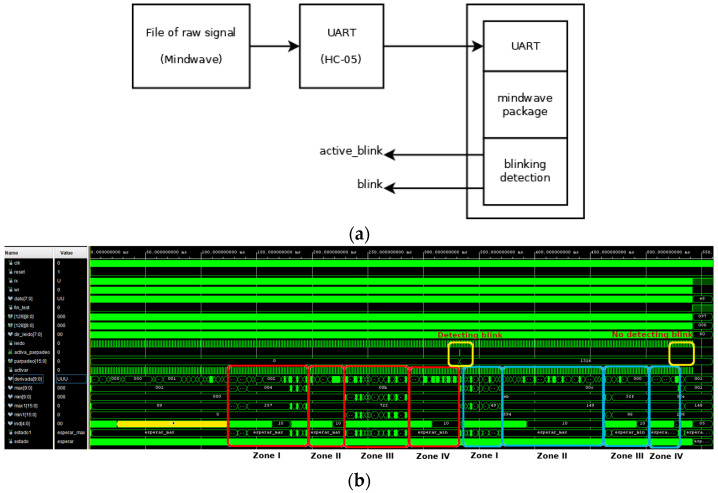
Simulation of the VHDL implementation of the algorithm for eye blinking detection. (**a**) Environment and (**b**) waveforms of the simulation (indicating the different zones used in the proposed algorithm and the difference between a blink and a rebound). Red boxes correspond to the main wave marked in red in Figure 4b, blue boxes correspond to the extra oscillation marked in blue in Figure 4b and, yellow boxes correspond to the results of algorithm.

The algorithm was directly implemented in VHDL, a hardware description language, and the simulation result is shown in Figure 9. The environment of the simulation is shown in Figure 9a. A file with the samples corresponding to the raw signal in Figure 4 was generated, capturing the information transmitted from the Mindwave headset. This file substitutes the headset in Figure 1. After that, this information was transmitted through a UART module, substituting the HC-05 device. Finally, the three described modules in the FPGA device were considered: the UART module, the controller of the mindset communication protocol, and the detection algorithm.

The waveforms corresponding to the simulation are shown in Figure 9b. Here, two artifacts are marked: one corresponding to the blink, marked in red (also marked in red in Figure 4b), and one corresponding to the rebound, marked in blue (also marked in blue in Figure 4b). In the first case, four zones can be distinguished. Firstly (zone one in Figure 4b), the algorithm seeks the maximum value (11 for the differentiated signal and 722 for the raw signal). After that, the algorithm seeks the edge between the maximum and minimum values (when the differentiated signal passes through 0, zone two in Figure 4b). Thirdly (zone three in Figure 4b), the algorithm seeks the minimum value (−21 for the differentiated signal and −594 for the raw signal). Finally, it seeks the end of the artifact in the fourth zone (when the differentiated signal reaches the value 0, zone four in Figure 4b). Since the maximum and minimum values pass their respective thresholds, the activation of the active_blink signal (marked in yellow) indicates the existence of a blink immediately.

The behavior of the rebound signal is similar to the previous one, with the same zones. Nevertheless, in this case, the minimum value (−2) does not pass its threshold and, therefore, the active_blink signal is not activated (marked in yellow), indicating the non-existence of a blink.

### 4.3. Comparison between e-Sense Value and Proposed Algorithm

In order to show the behavior of the proposed algorithm, the samples when an eye blink was detected were determined. The waveform in Figure 5 was used. For this, the software supported by Neurosky for Visual Studio (Microsoft, Redmond, WA, USA) was used, monitoring the raw signal and the blink strength (measure provided by the Neurosky software called e-sense data). Subsequently, this raw signal sequence was fed into a software version of the proposed algorithm.

It is worth mentioning that the Neurosky software seeks blinks through a significant variation in the average value of the raw signal. The average value of *N* − *N*1 samples is shown in Equation (2), which can be rewritten as in Equation (3) (subtracting the sample value corresponding to the *N* − *N*1 − 1 sample and adding the sample value corresponding to the *N* sample). It can be observed that Equation (3) is virtually the same as the differentiated value Equation (1) used in the proposed algorithm, removing the offset component.

(2) $$\mathrm{average}\left[N\right]=\frac{1}{N1}\overset{N1}{\underset{p=0}{\sum }}F\left[N-p\right]$$

(3) $$\mathrm{average}\left[N\right]=\mathrm{average}\left[N-1\right]+\frac{F\left[N\right]-F\left[N-N1-1\right]}{N1}$$

The result obtained is shown in Table 1. This shows the index of the sample when the Neurosky software detected the blink, the value of the blinking strength given by the Neurosky software, the index of the sample when the proposed algorithm detected the blink, the wave amplitude of the differentiated signal (amplitude one), and the amplitude of the raw signal (amplitude two). In most cases, the proposed algorithm detected the blink before the Neurosky software (except for the artifact p4). However, the difference between both measurements was about 20 samples. Considering that the frequency of the samples was 512 Hz, these 20 samples correspond to a time of about 40 ms, which is not significant in a human timing scale. With respect to the value of the blink, amplitude one and amplitude two show similar behavior to the e-sense value.

**Figure 10:**
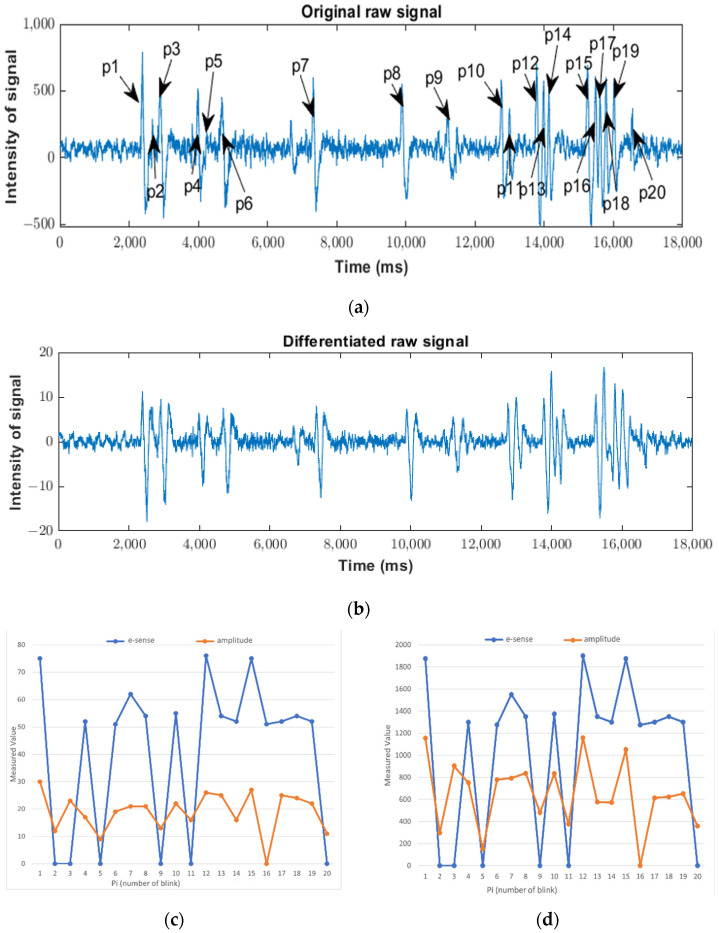
Comparison between e-sense value from Neurosky software and the amplitude of differentiated (**c**) and raw (**d**) signals. Additionally, this shows the waveforms of the raw (**a**,**b**) differentiated signals used in the comparison. p1–p20 indicate blinks in raw signal.

In order to obtain a more effective vision of the behavior of the values assigned to the blinks, the waveforms of raw and differentiated signals shown in Figure 10a,b were used. Here, different kinds of blinks were considered: a single blink, multiple blinks, strong blink, soft blink, etc. The comparison between the e-sense values, the amplitude of the differentiated signal, and the amplitude of the raw signal is shown in Figure 10c,d. In the case of Figure 10d, the e-sense values were scaled with a factor of 20 to be comparable with the amplitude of the raw signal. In these figures, a value of zero indicates that a blink was not detected.

Firstly, it is worth noting that the Neurosky software did not detect 6 artifacts out of 20; therefore, the error rate was about 30%, amounting to light blinks. However, the proposed algorithm did not detect 1 artifact out of 20 and, therefore, the error rate was about 5%. The undetected blink was within a multiple sequence of five blinks. In particular, that blink was filtered by the differentiated signal, as can be seen in Figure 10b.

Secondly, the signal with behavior that was more similar to the e-sense values was the amplitude of the raw signal. This may have been due to the larger range of values for this signal compared to the differentiated signal.

## 5. Results

The goodness of our algorithm lies in its use when performing a task that requires concentration, so we did not consider evaluating it under other conditions. Therefore, we conducted tests exclusively with data obtained in our laboratory, always considering intentional blinks. 

**Figure 11:**
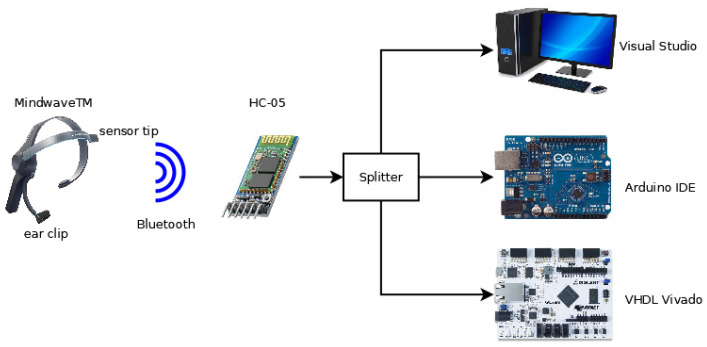
Environment of the experimental results.

Therefore, the proposed algorithm implemented on VHDL was compared with the Neurosky software and a software version on Arduino (Somerville, MA, USA) [38]. The environment used for the comparison is shown in Figure 11. The mindwave headset passed the information through the HC-05 module. The rx channel of this module was connected to three systems via a splitter: the FPGA device and the Arduino UNO through a connection to internal UART modules and the PC through an UART-USB converter. Therefore, it was warranted that the three systems would have the same input data at each moment for comparison.

The Neurosky algorithm is proprietary and, hence, we did not know its characteristics. The only information about it was that it is based on the adaptive average value of the raw signal. This software was executed using Visual Studio™.

The algorithm of the Arduino version seeks the average value of the raw signal each second; that is, every 512 samples. If this average value does not pass a certain threshold (which must be configured for each user), the algorithm considers that there is not a blink and is ready to detect the following blink. Otherwise, the system will wait until the average value is reduced below the early threshold.

The code for each version was lightly modified according to the following functionalities. We set the Visual Studio version to output the raw signal and the strength of eye blinking so that a waveform of the raw signal could be generated to visualize the different artifacts and to obtain the strength and the sample of the raw signal in which the blink was detected. We set the Arduino version to output the average of eye blinking and the sample in which this average passed a certain threshold. Finally, we set the VHDL version to output the amplitude of the raw signal corresponding to an eye blink and the sample in which the blink was detected. Additionally, a timer (with a precision of about 0.3 ms) was included in the VHDL version to identify the arrival time of each sample. The transmission speed was set to high (about 250,000 bauds) as the arrival of new data from the headset was affected.

Using these platforms, a total of ten different scenarios were considered. The main characteristics of each scenario are shown in Table 2, including the type of sequence, the number of artifacts, and the approximate duration time.

### 5.1. Study on the Efficiency of Detection Algorithms

**Figure 12:**
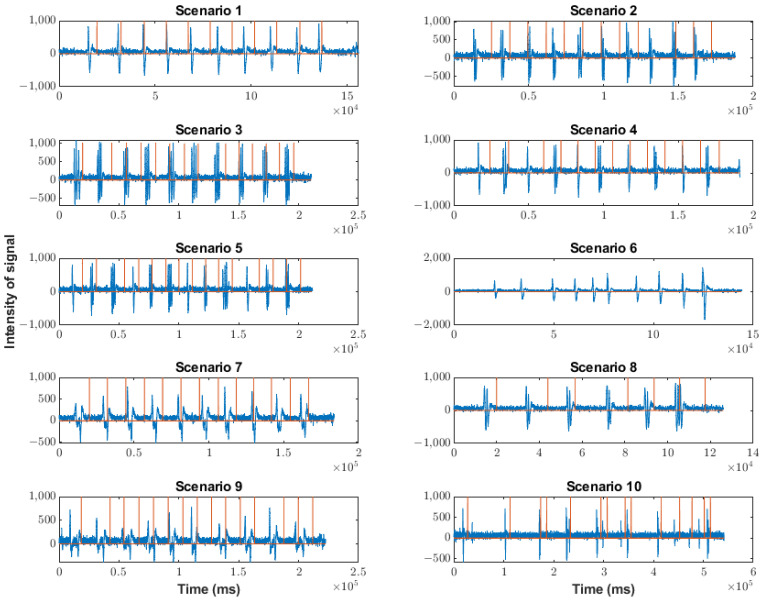
Behavior of Arduino version in the scenarios indicated in Table 2. Orange lines indicate a blink detection.

**Figure 13:**
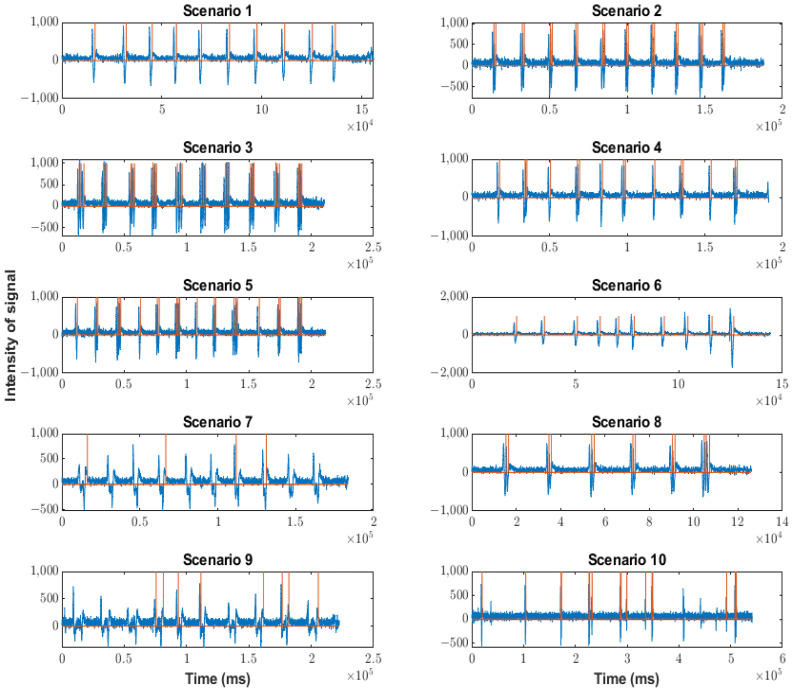
Behavior of Neurosky version in the scenarios indicated in Table 2. Orange lines indicate a blink detection.

**Figure 14:**
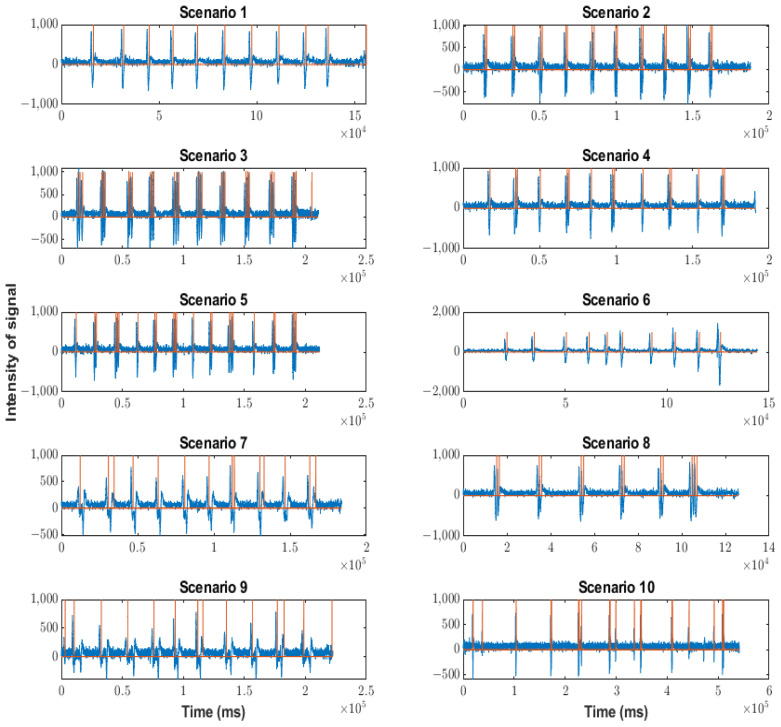
Behavior of FPGA version in the scenarios indicated in Table 2. Orange lines indicate a blink detection.

The behaviors of each version in the different scenarios are shown in Figure 12 (corresponding to the Arduino version), Figure 13 (corresponding to the Neurosky version), and Figure 14 (corresponding to the FPGA version). The blue waveforms are the raw signal and the orange waveforms indicate the existence of a blink (when they are different from zero). The x-axes indicate the arrival ticks (one tick per 0.3 ms) of each sample.

For the Arduino version, data were lost because the processing speed was low (16 MHz). This data loss could involve the loss of artifacts.

Additionally, it can be observed that there was no detection of multiple blinks. This was due to the operation of the detection, obtaining the average value of the raw signal in one second. Additionally, the detection threshold was too low because it detected an artifact when there was none. Therefore, the Arduino version was not considered in the following studies. Furthermore, the Arduino version suffered data loss due to a slow processing speed.

**Figure 15:**
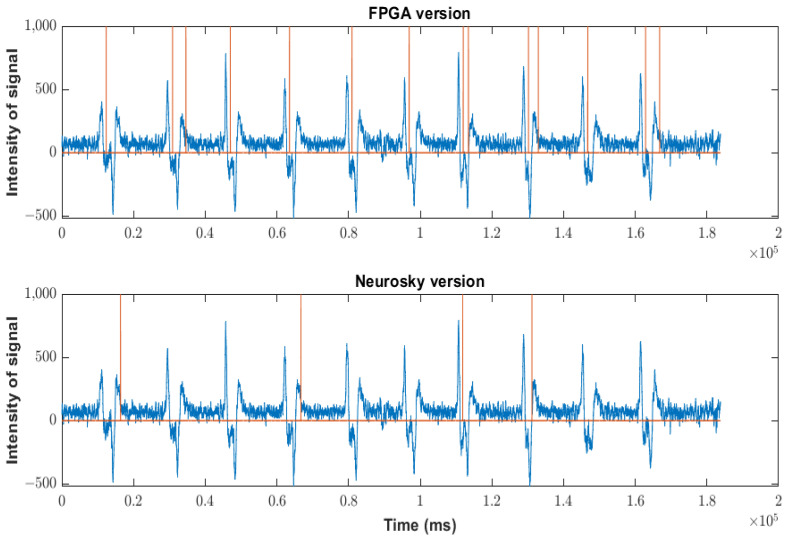
Behavior of FPGA version versus Neurosky version in scenario seven, showing how the rebound affects blinking detection. Orange lines indicate a blink detection.

The data loss in the Neurosky version was very low compared to the Arduino version, mainly due to the different processing speeds. However, it existed when the event rate increased, which did not happen appreciably with our algorithm in the FPGA, as shown below. Most blinks were detected, but when the sequence had artifacts of different kinds and strengths (as in the case of scenario 10), several blinks were not detected. Particularly, in the case of scenario 10, the non-detection rate was 3/21. Scenarios seven and nine were special because the shape of the soft blinks was different from that of the traditional blinks. As can be seen in Figure 13 and Figure 15, the shape showed a short rebound before reaching the minimum value. The effect of this rebound was the non-detection of the blink (50% of the cases) or the detection of a double blink (10% of the cases).

For the FPGA version, there was no data loss due to the parallel processing typical of hardware implementation.

The process of detection was more uniform than in the case of the Neurosky version. In fact, all artifacts were detected. The platform may also have detected artifacts considered non-intentional blinks because the amplitude was low. To avoid these detections, the threshold must simply be increased. In the case of scenarios seven and nine, the effect of the rebound was the detection of a double blink (30% of the cases). This effect can be reduced by increasing the detection threshold in the algorithm. In any case, as shown in Figure 15, the resolution of the algorithm implemented on the FPGA was more efficient, even in this complicated scenario. It is important to remember that soft blinks require some training by the user.

As mentioned above, the soft blink waveform is different from that generated by a normal blink. It also occurred in scenario nine, when eyes were closed for a second, and, hence, scenarios seven and nine were not considered in the following studies.

### 5.2. Study on the Detection Time of Blinks

**Figure 16:**
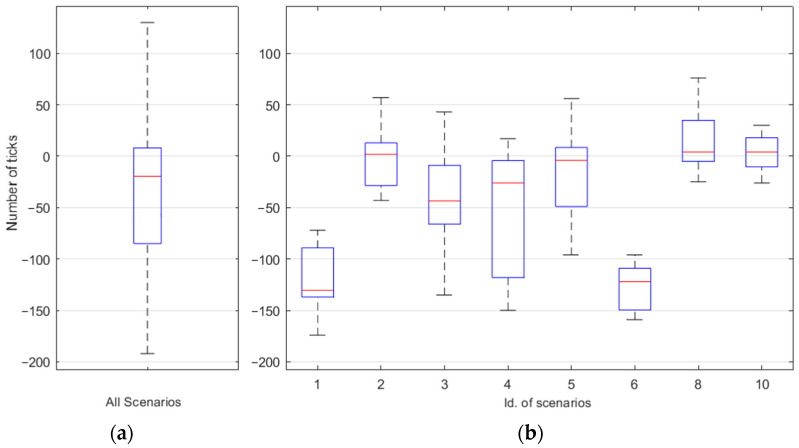
Difference (in number of ticks) between blinking detection times for the scenarios indicated in Table 3. (**a**) Difference considering all scenarios. (**b**) Difference in each scenario.

One of the characteristics to study was the detection time. The detection time was measured through the difference in the arrival ticks corresponding to the samples prior to the blinking value. These ticks were translated to time (because each tick corresponded to approximately 0.3 ms in accordance with the timer implemented in the FPGA device). The difference was the tick of the Neurosky version minus the tick of the FPGA version. Therefore, a negative difference implied that the detection with the FPGA version was faster than with the Neurosky version. Figure 16 and Table 3 show the difference between the ticks corresponding to the Neurosky and FPGA versions.

In most cases, the FPGA version detected the blink before the Neurosky version. This means that the FPGA version requires fewer samples to perform detection. Overall, the FPGA version required 19.5 fewer samples than the Neurosky version, which required 6.39 ms.

### 5.3. Study on the Strength of Blinks

**Figure 17:**
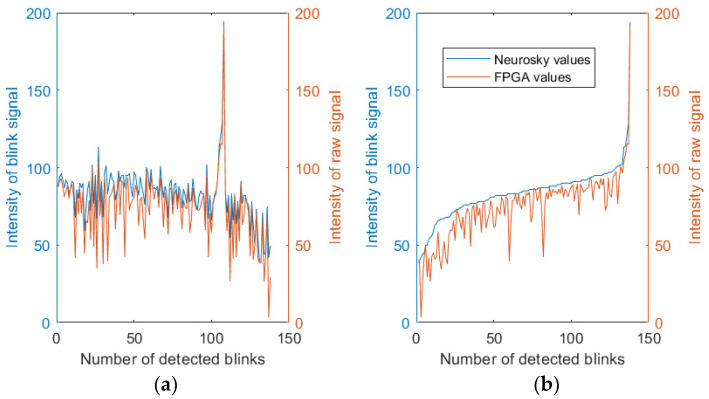
Values of adapted blinking strength obtained for all artifacts found in the scenarios indicated in Table 3. (**a**) Artifacts ordered by their identification. (**b**) Artifacts ordered by the strength value from the Neurosky software.

Although the proposed algorithm discards most non-intentional blinks, it is usually necessary to measure the strength of the blink to improve the threshold provided by the algorithm or to use different values of blink intensity to generate different actions. Figure 17 shows the strength values corresponding to the detection with the Neurosky version (in blue) and the FPGA version (in orange). All artifacts detected by both versions in the scenarios included in Table 3 were used, totaling 138 artifacts. These artifacts were ordered in two different manners. Firstly, in Figure 17a, the artifacts were ordered according to their detection (firstly, the artifacts in scenario 1, and lastly, the artifacts in scenario 10). An adaptation factor was studied to reduce the scale factor between both versions, resulting in the variation in strength values being very similar. Secondly, in Figure 17b, the artifacts were ordered by the strength value indicated by the Neurosky version.

Again, it can be proved that the tendencies for the values in both versions were the same. Therefore, it can be concluded that both measurements were equivalent.

## 6. Conclusions

This study focused on detecting eye blinks using a single-channel BCI system (a Mindwave headset from Neurosky). The study found that the blinks had characteristic maximum and minimum values in the same slope, which were used to propose a detection algorithm. The algorithm can be configured to adjust the detection sensitivity and detect blinks of varying strengths.

The proposed algorithm was implemented in an FPGA device and compared to two versions: the system provided by Neurosky and a version implemented with a platform based on Arduino. The comparison was based on three parameters: efficiency, detection time, and blinking strength.

In terms of efficiency, the study showed that the FPGA version did not lose any samples due to the parallel processing of the hardware implementation, while data loss occurred in the Arduino version and in some scenarios in the Neurosky version. In terms of error rate, the Arduino version had a high rate due to the difficulty in estimating the average value of the raw signal, while the Neurosky version had an error rate when the sequence included blinks of different types and strengths. The FPGA version detected unintentional blinks in addition to intentional ones, but this could be easily reduced by increasing the detection thresholds. In terms of detection time, the FPGA version was faster than the other versions, requiring fewer samples to perform the detection. Finally, in terms of blink strength, the proposed algorithm showed equivalent results compared to the proprietary BCI headset, which implies that, overall, we have an efficient, fast, and effective algorithm that allows us to use signals generated by intentional eye blinks as control signals in human–machine interface systems.

## Figures and Tables

**Table 1:** Comparison between e-sense values and proposed algorithm.

	N. Sample for Raw Signal		N. Sample for Differentiated Signal		
	Index	e-Sense Value	Index	Amplitude One	Amplitude Two
P1	1888	51	1881	18	820
P2	2674	74	2669	27	1109
P3	3516	80	3501	30	1162
P4	4118	73	4125	27	1147
P5	4223	65	4221	28	781

**Table 2:** Main characteristics of each scenario used in the comparison. The characteristics are the type of sequence, the number of artifacts included, and the approximated duration of time.

Scenario	Type of Sequence	N. of Artifacts	Time (s)
1	Sequence of 10 simple blinks	10	25
2	Sequence of 10 double blinks	20	30
3	Sequence of 10 triple blinks	30	35
4	Sequence of 10 blinks, alternating simple and double	15	31
5	Sequence of 12 blinks, alternating simple, double, and triple	24	35
6	Sequence of 10 simple blinks, increasing in strength	10	24
7	Sequence of 10 very soft blinks	10	30
8	Sequence of five double blinks plus a triple blink	13	20
9	Sequence of 10 simple blinks, maintaining the eye closed for approximately one second	10	37
10	Natural sequence including different kinds of blinks	21	89

**Table 3:** Difference (in number of ticks and ms) between blinking detection times.

Scenarios	1	2	3	4	5	6	8	10	Total
Difference (n. of ticks)	−130.5	2	−43.5	−26	−4	−122	4	4	−19.5
Difference (ms)	−42.7	0.7	−14.3	−8.5	−1.3	−39.98	1.3	1.3	−6.39

## Data Availability

Data is available on request.
